# Evaluation of antioxidant and anticancer activities of naphthoquinones‐enriched ethanol extracts from the roots of *Onosma hookeri* Clarke. var. longiforum Duthie

**DOI:** 10.1002/fsn3.1729

**Published:** 2020-06-29

**Authors:** Qiang Wu, Aga Er‐bu, Xiaoxia Liang, Shangxian Luan, Yue Wang, Zhongqiong Yin, Changliang He, Lizi Yin, Yuanfeng Zou, Lixia Li, Xu Song

**Affiliations:** ^1^ Natural Medicine Research Center College of veterinary medicine Sichuan Agricultural University Chengdu P. R. China; ^2^ Medical college Tibet University Lasa P. R. China

**Keywords:** anticancer, antioxidant, naphthoquinones‐enriched ethanol extract, *Onosma hookeri* Clarke. var. longiforum Duthie

## Abstract

In this study, the optimal naphthoquinones‐enriched ethanol extract from the roots of *Onosma hookeri* Clarke. var. longiforum Duthie (*OHC*‐LD) was obtained under an optimal condition (69% ethanol, material to solution ratio of 27:1 at 60℃ for 59 min) by the ultrasound‐assisted extraction, according to four‐variable three‐level Box–Behnken design‐response surface methodology. The experimental yield of ethanol extract was 42.08 ± 0.65%, and the contents of naphthoquinones reached to 1.07 ± 0.004%. The optimal extract exhibited similar scavenging activity against ABTS (2,2'‐azino‐bis‐3‐ethylbenzthiazoline‐6‐sulfonic acid) radical as BHT(butylated hydroxytoluene) at 1,250 µg/ml, and better DPPH (2,2‐diphenyl‐1‐picrylhydrazyl) scavenging activity than BHT at 250 µg/ml. However, the optimal ethanol extract was not sensitive to MCF‐7 cell line ( IC_50_ of 321.849 µg/ml). The results revealed the naphthoquinones‐enriched ethanol extract from the roots of *OHC*‐LD had could be used as a potential natural antioxidant.

## Graphical abstract

Optimization of the ultrasound‐assisted extraction of naphthoquinones‐enriched ethanol extracts from Onosma hookeri Clarke. var. longiforum Duthie using response surface methodology. Anticancer and antioxidant activities of alcoholic extracts from Onosma hookeri Clarke. var. longiforum Duthie. Increased naphthoquinones content in alcohol extracts by process optimization.

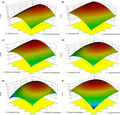

## 1 INTRODUCTION

“Zicao,” a traditional Chinese medicine for various skin conditions and injuries, has been used for a long history (Papageorgiou, Assimopoulou, Couladouros, Hepworth, & Nicolaou, 1999). Modern pharmacological studies indicated its extensive biological activities, including anti‐inflammation (KaithKaith & Chauhan, 1996; Weng et al., 2000), antibacterial (Sasaki, Yoshizaki, & Abe, 2000), antifungal (Sasaki, Abe, & Yoshizaki, 2002), antiviral (e.g., human immuno‐deficiency virus, varicella‐zoster virus, and influenza virus) (Xin et al., 2003; Yamasaki et al., 1993), and antitumor (Yutaka et al., 2004). However, the source of “Zicao” is not only from the roots of *Lithospermum erythrorhizon* Sieb. et Zucc. (*L. erythrorhizon*), although most of the reports have been focused on it. Other resources, such as *Onosma hookeri* Clarke. var. longiforum Duthie (*OHC*‐LD), which has been widely used in Tibet medicine as the replacement of *L. erythrorhizon*, has not been investigated deeply.

Basically, naphthoquinones have been considered as their active constituents (Lei et al., 2009). The main naphthoquinone compounds from “Zicao,” such as shikonin, acetylshikonin, and β, β‐dimethyl‐acrylshikonin, as well as the extracts from *L. erythrorhizon,* have been reported as excellent antioxidants (Weng et al., 2000; Zhang, Yang, & Zhu, 2007). Recently, six main naphthoquinone compounds, such as shikonin, acetylshikonin, β‐acetoxy‐isovalerylshikonin, isobutyrylshikonin, β,β‐dimethylacryl‐ shikonin, and 2‐methylbutyrylshikonin, have also been isolated from the roots of *OHC*‐LD (Li et al., 2014; Zan et al., 2016). All the isolated main naphthoquinones‐compounds are shikonin derivatives, which have exhibited excellent antioxidant capacity (Ordoudi et al., 2011). Moreover, shikonin and its derivatives have been reported as inhibitors against MCF‐7 cells (Human breast cancer cells) by targeting cell necroptosis and apoptosis (Deniz, Ibis, et al., 2015; Deniz, Mustafa, Ayse, & Resat, 2015; Shahsavari, 2018), indicating its anticancer potential against human breast cancer. However, an appropriate extraction technology largely determines whether its antioxidant or anticancer activity could reach to a high level or not.

Therefore, in this study, an naphthoquinones‐enriched ethanol extracts from the roots of OHC‐LD was conducted by the ultrasonic‐assisted technology (Hao, Li, & Lin, 2017; Jovanović et al., 2017) and the Box–Behnken design‐response surface methodology (BBD‐RSM) (Liu, Miao, Wen, & Sun, 2009; Ye & Jiang, 2011; Yue, Shao, Yuan, Wang, & Qiang, 2012). Besides, the antioxidant capacity of the optimal ethanol extract was measured by scavenging activity against both ABTS (2,2'‐azino‐bis‐3‐ethylbenzthiazoline‐ 6‐sulfonic acid) radical and DPPH (2,2‐diphenyl‐1‐picrylhydrazyl) radical, the reducing power, and the antitumor activities by MTS (3‐(4,5‐dimethylthiazol‐2‐yl)‐5 (3‐carboxy‐methoxyphenyl)‐2‐(4‐sulfopheny)‐2H‐tetrazolium) method. It would be useful for its exploitation in the future.

## 2 MATERIALS AND METHODS

### 2.1 Plant materials

The roots of *OHC*‐LD were collected from Shigatse, Tibet and authenticated by Aga Er‐bu of Tibet University. The voucher specimen (No. 20171216‐1) has been deposited at Pharmacy Department, Sichuan Agricultural University.

### 2.2 Chemical reagents

DPPH (purity greater than 97%) was purchased from BioDukly, and ABTS (purity greater than 98%) was obtained from Solarbio. MTS (Promega), BHT (butylated hydroxytoluene), Dimethyl sulfoxide (DMSO), and Adriamycin were obtained from SERbio. All other chemical reagents were of analytical grade and obtained from Kelong Chemical Co. Lt).

### 2.3 Cell lines and culture medium

MCF‐7 cells were purchased from Shanghai Cell Bank, Chinese Academy of Sciences. MCF‐7 cells were cultured in 5% CO_2_, for 24 hr at 37℃ in an incubator (Thermo). The growing medium consisted of the following: Roswell park memorial institute (RPMI) 1640 medium (HyClone), 10% Fetal bovine serum (Gibco), 2% antibiotics [penicillin in a concentration of 100 U/mL and streptomycin in a concentration of 100 U/mL (Gibco)], and 1% insulin (Gibco).

### 2.4 Preparation of the naphthoquinones‐enriched ethanol extract by Ultrasonic‐assisted extraction according BBD‐RSM

The roots of *OHC*‐LD (2 g) were added ethanol solution (55%–95%) with the ratio of material to solution of 1:10 to 1:30 g/ml into a 100 ml conical flask. The ethanol extract was extracted by ultrasonic‐assisted extraction at 20℃ to 70℃ for 20 to 70 min. After being centrifuged at 3,500 rpm for 5 min, the supernatant was collected and made up to 50 ml, 10 ml of which was concentrated by a rotary evaporator under vacuum at 50℃ and dried to constant weight for the measurement of its total weight. The content of naphthoquinones was calculated by the UV absorption value of L‐shikonin at 516 nm. A standard curve was prepared using L‐shikonin (Y = 18.495X + 0.0257 R^2^ = 0.9991, Linearity range, 4 μg/ml to 44 μg/ml). The contents of naphthoquinones were expressed as micrograms of L‐shikonin per gram of sample.

#### 2.4.1 Single‐factor experiment

During ultrasonic‐assisted extraction of the roots from *OHC*‐LD, the effects of extraction temperature and time, the ratio of material to solution, and ethanol concentrations were investigated by a single‐factor design. Each experiment was done when one factor was changed, while the others were remained (Chen et al., 2017). The effects of each factor were analyzed by the total weight of ethanol extract and its content of naphthoquinones as Equation (1):

(1) $$\text{Comprehensive score}=\frac{X}{X_{\max}}\times 50+\frac{Y}{Y_{\max}}\times 50$$

where *X*_max_ represents the highest extraction rate of naphthoquinones in all experimental groups, X represents the extraction rate of naphthoquinones for each experiment; Where *Y*_max_ represents the highest weight of ethanol extract in all experimental groups, *Y* represents the weight of ethanol extract for each experiment.

#### 2.4.2 BBD‐RSM experimental design

A BBD‐RSM was designed by a commercial statistical package, Design‐Expert version 8.0.6, to estimate the effect of each independent variables (extraction temperature and time, the ratio of material to solution, and ethanol concentration) basing on both the extraction yields and the contents of naphthoquinones. According to the results of single‐factor experiments, each parameter experiment was performed on three different levels, see in TABLE 1. In this study, the design consisted of 29 experimental points, including five center points to calculate the repeatability of the method (Noshad, Mohebbi, Shahidi, & Mortazavi, 2012). In order to minimize the effects of unexplained variability, experiments were randomized. Then, the variables were calculated by the Equation (2):

(2) $$X=\frac{X_{i}-X_{0}}{\Delta X}$$

where *X* is a coded value for the variable, X_i_ is the corresponding actual value, *X*_0_ is the actual value in the center of the domain, and Δ*X* is the step change value.

**TABLE 1:** Independent factors and their levels used in ultrasonic extraction

Independent factors	symbol	Levels		
		−1	0	1
extraction temperature (℃)	A	40	50	60
extraction time (min)	B	50	60	70
liquid–solid ratio(v/m)	C	20	25	30
ethanol concentration（%）	D	55	65	75

The scoring of extraction yields and the contents of naphthoquinones were calculated via second‐order polynomial equation as Equation (3):

(3) $$Y=A_{0}+\sum_{i=1}^{4}A_{i}X_{i}+\sum_{i=1}^{4}A_{\mathrm{ii}}X_{i}^{2}+\sum_{i=1}^{3}\sum_{j=1}^{4}A_{\mathrm{ij}}X_{i}X_{j}$$

where *Y* represents the dependent variable (scores of yields of ethanol extract and the contents of naphthoquinones); A_0_ is the constant coefficient; *A_i_*, *A_ii_*_,_ and *A_ij_* are the coefficients estimated by the model; *X_i_* and *X_j_* are the coded independent variables. They represent the linear, quadratic, and interaction effects of the *X*_1_, *X*_2_, *X*_3,_ *X*_4_ factors, respectively; (i ≠ j) (Noshad et al., 2012).

### 2.5 Antioxidant activity

The antioxidant capacity of the crude extract, which was obtained under the optimal conditions, was investigated by DPPH method, ABTS method, and reduction powder method.

#### 2.5.1 The radical scavenging activity against DPPH

The radical scavenging activity of the ethanol extract against DPPH was according to the literature (Li, Hao, Wang, Huang, & Li, 2009). In brief, the mixture of DPPH solution (1 ml, 0.048 mg/ml in 75% ethanol) and the ethanol extract solution (1 ml, 3.91–1000 µg/ml) was incubated at 20℃ in the dark for 30 min. Then, the absorbance was measured at 517 nm. The butylated hydroytoluene (BHT) was used as a positive control. Equation (4):

(4) $$\text{The DPPH radical scavenging activity}\left(\%\right)=\left(1-\frac{A_{i}-A_{j}}{A_{0}}\right)\times 100\%$$

where *A_i_* represents the absorbance of the DPPH/sample mixture, *A_0_* represents the absorbance of the DPPH without sample (the sample was replaced by 75% ethanol), and A*_j_* represents the absorbance of the sample without DPPH (the DPPH was replaced by 75% ethanol).

#### 2.5.2 The radical scavenging activity against ABTS

The radical scavenging activity against ABTS was measured as followed: the 1:1 (v/v) mixture of K_2_S_2_O_2_ solution (2.6 mmol/L) with ABTS (7.4 mmol/L) solution was kept in the dark at room temperature for 12–16 hr. The ABTS working solution (0.8 ml), which was obtained by diluting the mixture above with absolute ethanol, was mixed with ethanol extract solution (0.2 ml, 19.53–5000 µg/ml). Taking BHT as a positive control, the absorbance of the sample was determined at 734 nm (Li, Lin, Gao, Han, & Chen, 2012). Equation (5):

(5) $$\text{The ABTS radical scavenging activity}\left(\%\right)=\left(1-\frac{A_{i}-A_{j}}{A_{0}}\right)\times 100\%$$

where A*_i_* represents the absorbance of the ABTS/sample mixture, A*_0_* represents the absorbance of the ABTS without sample (the sample was replaced by 75% ethanol), and A*_j_* represents the absorbance of the sample without ABTS (the ABTS was replaced by 75% ethanol).

#### 2.5.3 Reducing power

The Reducing power was measured according to the literature (Oyaizu, 1986). The mixture of the ethanol extract solution (1 ml, 19.53–10000 µg/ml), phosphate buffer (2.5 ml, 0.2 mol/L, pH = 6.6), and potassium ferricyanide solution (2.5 ml, 10 g/L) was incubated at 50℃ under water bath for 30 min and then added trichloroacetic acid (2.5 ml, 100 g/L), centrifuged at 3,000 r/min for 10 min. The supernatant (2.5 ml) was mixed with distilled water (2.5 ml) and FeCl_3_ (0.5 ml, 0.1% w/v). After incubating at room temperature for 10 min, the absorbances of the sample mixture and BHT were measured at 700 nm.

### 2.6 Anticancer activity

The cytotoxic activity of the optimal ethanol extract was measured in vitro against MCF‐7 cell line (Breast cancer cell) using the MTS assay (Alaklabi, Arif, Ahamed, Radhakrishnan, & Akbar, 2017). The ethanol extract was dissolved in DMSO. Taking DMSO as blank contrast, control cells were treated with Adriamycin. Cultivate tumor cells were seeded in each well of the 96‐well plates with 1 × 10^5^ cells/well. After incubation with the optimal ethanol extract with the appropriate concentration ranges of drugs (150, 75, 37.5, 18.75, and 9.375 µg/ml) for 48 hr, MTS solution (2.5 mg/ml in PBS) was added (20 μl/well), and the plates were incubated for an additional 2–4 hr at 37℃. The optical density of the solution was measured at 490 nm using a microplate reader (TECAN, Austria). Growth inhibition was estimated from the optical density of the solution. The percentage of cell survival was calculated as follows. Equation (6):

(6) $$\text{Survival fraction}=O.D.\left(\text{treated cells}\right)/O.D.\left(\text{control cells}\right).$$

The relation between surviving fraction and compound concentration was plotted, and IC_50_ (the concentration required for 50% inhibition of cell viability) was calculated for each test compound.

### 2.7 Statistical and analysis

All experiments were performed in triplicate, all data were expressed at least 3 independent evaluations, and standard deviation (*SD*) were also calculated using SPSS 23.0.

## 3 RESULTS AND DISCUSSION

### 3.1 Preparation of the ethanol extract

In order to achieve the highest extraction yields as well as the better contents of naphthoquinones, the effects of extraction temperature and time, the ratio of material to solution and ethanol concentration were investigated.

#### 3.1.1 Optimization of extraction process

**FIGURE 1:**
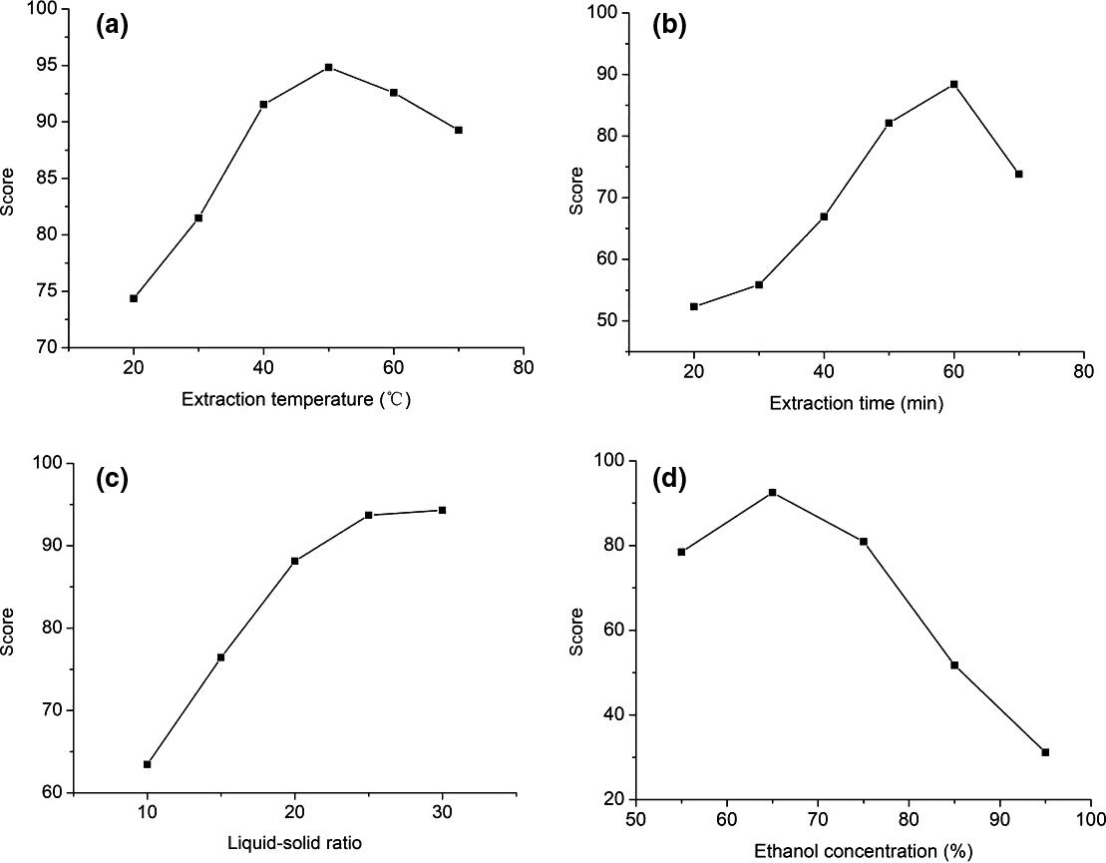
Scoring of the extraction yields and naphthoquinones content in single‐factor experiments: extraction temperature (A), extraction time (b), liquid–solid ratio (c), and ethanol concentration (d)

As shown in Figure 1a, the comprehensive score increased with temperature increasing from 20℃ to 50℃, reached maximum at 50℃, and decreased from that point. Thus, the favorable extraction temperature was 50℃.

As shown in Figure 1b, the highest comprehensive score was obtained when the extraction time was 60 min. After that point, the scores decreased. It may due to the degradation of naphthoquinones for over‐time extraction. Consequently, 60 min was the best extraction time.

As shown in Figure 1c, with the increase of the ratio of material to solution, the comprehensive scores were also increased. However, the increase was not obvious after the rate of 1:25. Considering the cost in the extraction process (Shao, Deng, Shen, Fang, & Zhao, 2011), 1:25 was chosen as the most favorable ratio of material to solution.

As shown in Figure 1d, the comprehensive scores increased along with ethanol concentration increase, and the highest score was obtained when the ethanol concentration was 65%. Accordingly, the favor the ethanol concentration ratio was 65%.

Therefore, the ethanol concentration of 55%–75%, extraction temperature of 40℃–60℃, the ratio of material to solution 20:1–30:1, and extraction time of 50–70 min were chosen as conditions in the optimization experiments (see Table 1).

#### 3.1.2 *Statistical analysis and model fitting using* response surface methodology (RSM)

Compared with traditional single parameter optimization, RSM is more advantageous for saving time, space, and raw material. For optimizing the four independent parameters, a total of 29 runs were in the current Box–Behnken design (see in Table 2). The fitting equation used to predict the extraction yield was as follows Equation (7):

(7) $$\begin{array}{cc} Y & =+96.43+2.88A+0.26B+4.40C+4.55D+0.46AB\\ & \;-0.018AC+1.09AD-1.00BC-3.42BD-1.86CD-\\ & \;1.04A^{2}-7.07B^{2}-3.86\;C^{2}-6.01D^{2} \end{array}$$

where *Y* represents the scores of naphthoquinones extract and alcohol extract, and *A*, *B*, *C*, and *D* are the coded variables for the extraction temperature, extraction time, the ratio of material to solution, and ethanol concentration, respectively.

**TABLE 2:** Box–Behnken design for independent variables and observed responses

Run no.	Extraction temperature (℃)	Extraction time (min)	Liquid–solid ratio	Ethanol concentration(%)	Alcohol extract (%)	Naphthoquinones(%)	score
1	0	−1	0	−1	34.70	0.753	75.44
2	0	0	0	0	40.18	1.099	97.10
3	0	−1	1	0	39.50	0.996	91.83
4	−1	0	−1	0	36.62	0.902	84.22
5	0	0	1	1	37.61	1.113	94.53
6	−1	0	0	1	37.69	1.008	90.08
7	−1	1	0	0	38.18	0.949	88.17
8	0	0	0	0	39.65	1.094	96.22
9	0	1	0	1	34.28	0.950	83.37
10	0	0	0	0	39.70	1.054	94.57
11	0	0	0	0	39.63	1.124	97.51
12	0	1	−1	0	34.72	0.867	80.37
13	1	1	0	0	38.55	1.098	95.02
14	0	0	−1	−1	35.62	0.756	76.69
15	1	0	0	−1	38.84	0.873	85.71
16	−1	0	1	0	39.60	0.974	91.03
17	0	−1	0	1	37.11	1.074	92.19
18	1	0	1	0	38.45	1.164	97.75
19	−1	−1	0	0	36.76	0.902	84.38
20	0	0	1	−1	40.26	0.927	89.79
21	1	−1	0	0	38.92	0.955	89.37
22	0	1	0	−1	36.02	0.829	80.31
23	0	0	−1	1	35.33	1.048	88.86
24	0	−1	−1	0	36.61	0.794	79.57
25	1	0	−1	0	37.09	1.047	91.01
26	0	1	1	0	38.63	0.947	88.63
27	−1	0	0	−1	38.08	0.838	83.30
28	0	0	0	0	39.37	1.114	96.73
29	1	0	0	1	38.61	1.139	96.85

The experimental data were analyzed by the Analysis of Variance, and the significance of the regression coefficients was evaluated by their corresponding P‐values (Ahmad, Alkharfy, Wani, & Raish, 2015). As shown in TABLE 3, the model difference was extremely significant (*p*‐value < .0001), and the Lack of Fit was not significant (*p*‐value = .1139 > .05). The R^2^ (0.9568) proved a reasonable fit between the model and the experimental data. The R^2^_adj_ (0.9136) also indicated the accuracy of the model. At the same time, three linear coefficients (*A*, *C*, and *D*), one interactive coefficients (BD), and three quadratic coefficients (*B^2^*, *C* ^2^, and *D* ^2^) were extremely significant (*p* < .01), and others were not significant (*p* > .05), indicating that the results were statistically significant. The results also indicated that the four factors of the test were not a simple linear relationship with the response value, but a result of multiple factors.

**TABLE 3:** Analysis of variance of response surface quadratic model analysis for the extraction yield

Source	Sum of squares	Degrees of Freedom	Mean squares	*F*‐value	*p*‐value prob > F
Model	1,156.4	14	82.6	22.16	<.0001^a^
A	99.36	1	99.36	26.66	.0001^a^
B	0.8	1	0.8	0.21	.6512
C	232.67	1	232.67	62.42	<.0001^a^
D	248.79	1	248.79	66.74	<.0001^a^
AB	0.86	1	0.86	2.30E−001	.6375
AC	1.23E‐003	1	1.23E‐003	3.29E−004	.9858
AD	4.75	1	4.75	1.27	.2778
BC	4	1	4	1.07	.3178
BD	46.85	1	46.85	12.57	.0032^a^
CD	13.8	1	13.8	3.7	.0749
A^2^	6.99	1	6.99	1.87	.1925
B^2^	324.04	1	324.04	86.93	<.0001^a^
C^2^	96.86	1	96.86	25.98	.0002^a^
D^2^	234.23	1	234.23	62.84	<.0001^a^
Residual	52.19	14	3.73		
Lack of Fit	46.98	10	4.7	3.61	.1139^b^
Pure Error	5.21	4	1.3		
Cor Total	1,208.59	28			

^a^ *p* < 0.05 Significant.

^b^ *p*> 0.05 Not significant.

**FIGURE 2:**
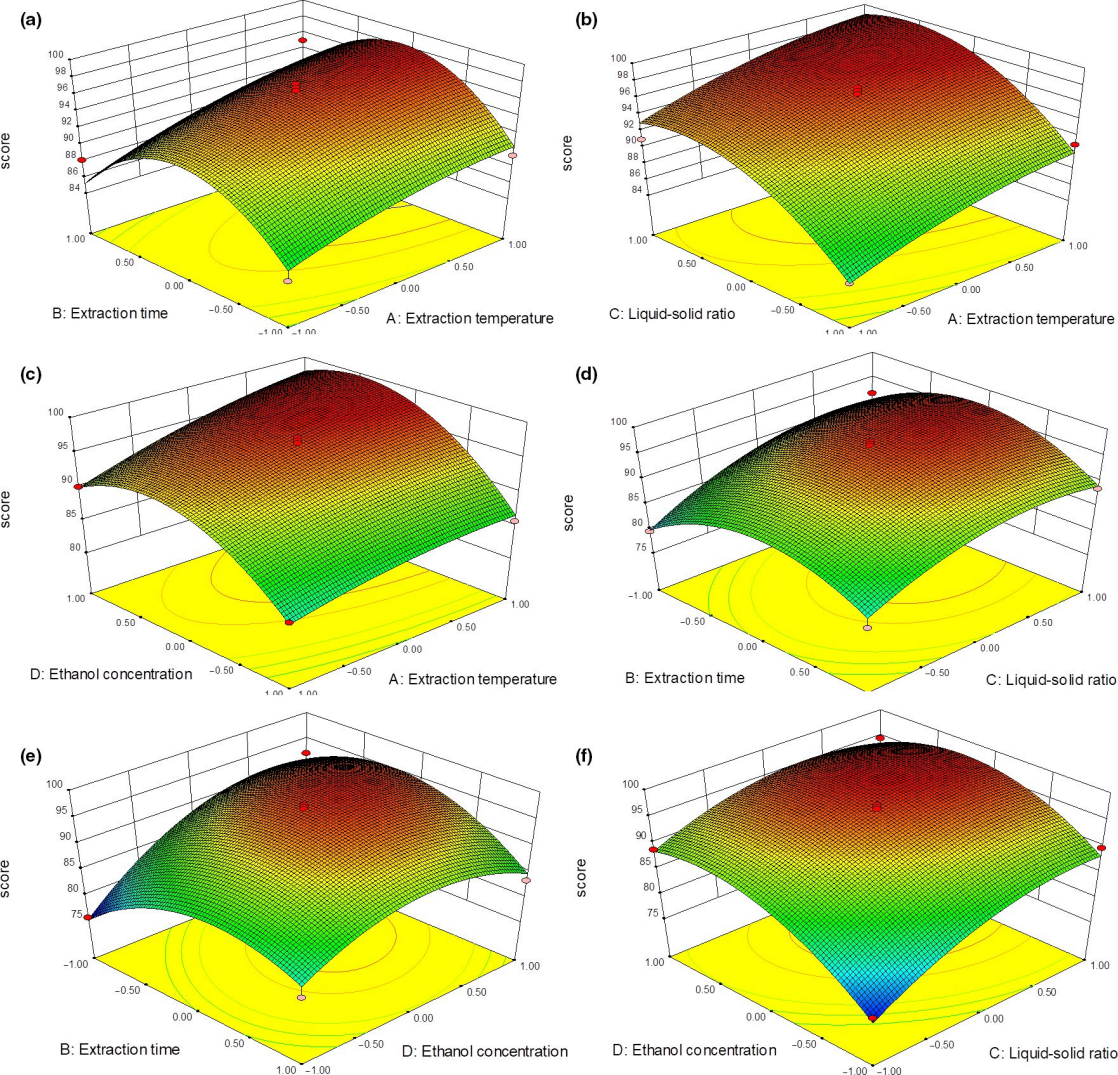
The 3D response surface maps showing the effects of two variables (the other two variables remain at zero in the analysis) on Grading of the yields of alcohol extract and naphthoquinones: significant interaction between (a) extraction time and extraction temperature (b) liquid–solid ratio and extraction temperature (c) ethanol concentration and extraction temperature (d) liquid–solid ratio and extraction time (e) extraction time and ethanol concentration, and (f) ethanol concentration and liquid–solid ratio

In the four‐factor three‐level response surface experiment, the response graph was a three‐dimensional graph (Figure 2), which fixed two factors and drew the influence of two other factors on the response value according to the fitting equation. The three‐dimensional map could intuitively reflect the influence of the interaction of these two factors on the response value. After the fitting equation of all the experimental data by Equation (7), the best process parameters were obtained: extraction temperature of 60℃, extraction time of 59.2 min, the ratio of material to solution of 27.4:1, and ethanol concentration of 69.2%.

#### 3.1.3 Verification of analytical method and the content of naphthoquinones in the optimal extraction

Three repeated verification tests were conducted under the optimal conditions, in which the sample was extracted with 69% ethanol with the ratio of material to solution of 27:1 at 60℃ for 59 min. Under these conditions, the extraction yield of ethanol extract was 42.08 ± 0.65% and the content of naphthoquinones reached to 1.07 ± 0.004%. The comprehensive score was 98.04 ± 0.89, which is close to the predicted value (100.467). The agreement of the results indicated that the experimental model had a good reliability, and the experimental value has little error. Therefore, an effective ultrasonic‐assisted extraction technique was optimized.

### 3.2 *Evaluation of antioxidant activities of the ethanol extract in vitr*o

#### 3.2.1 The radical scavenging activity against DPPH

**FIGURE 3:**
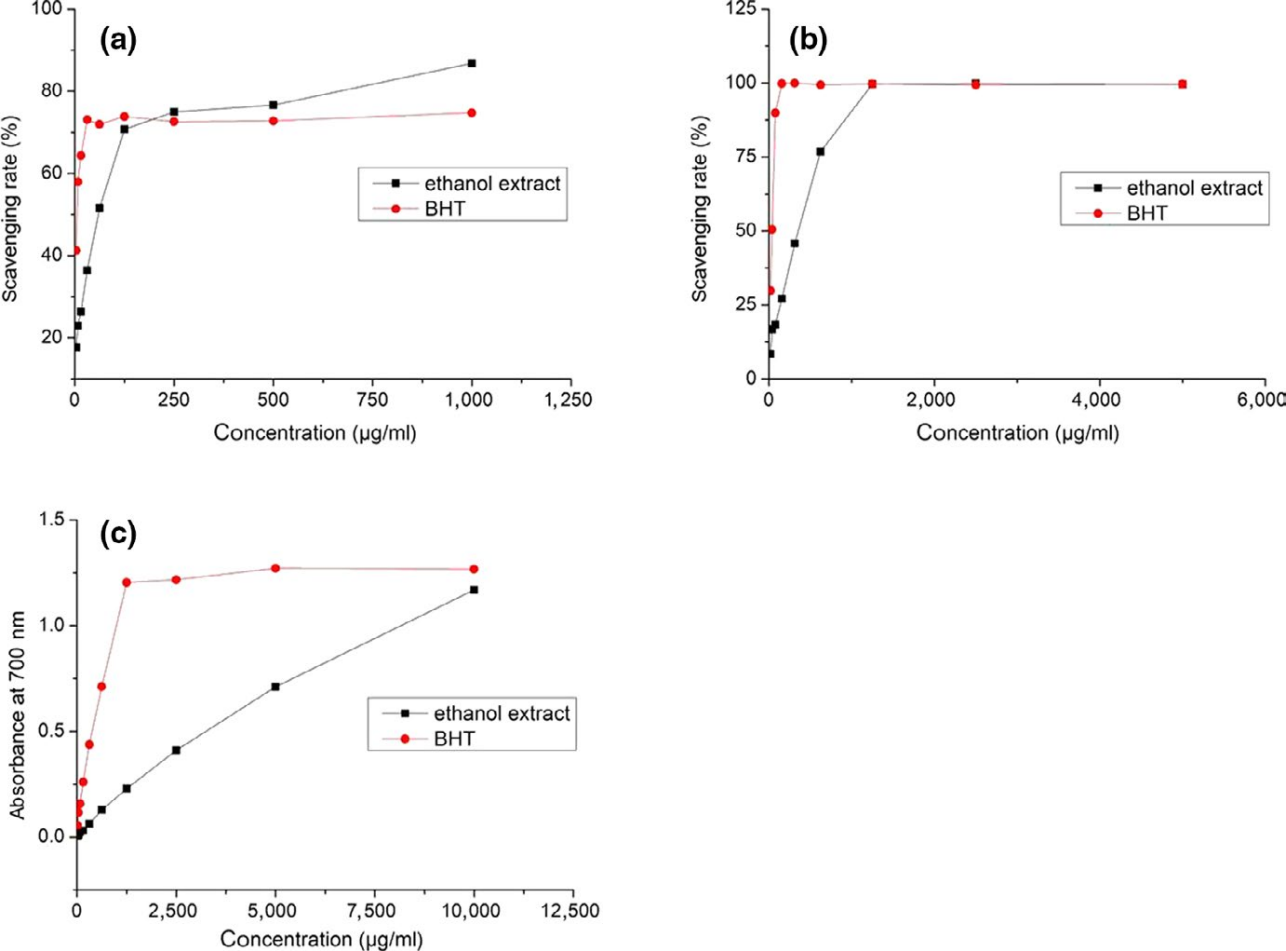
DPPH (a) and ABTS (b) radicals‐scavenging activities and Reducing power (c) of the ethanol extract in various concentrations. BHT was used as positive controls

As a free radical with an unpaired electron, DPPH could decrease significantly when it is exposure to proton radical scavengers (Rostami & Gharibzahedi, 2017). The DPPH scavenging activities of the ethanol extract were shown in Figure 3a, which exhibited dose‐dependent scavenging activities in the studied concentration range, with IC_50_ value of 51.30 µg/ml, higher than that of BHT (IC_50_ = 4.90 µg/ml). The BHT achieved maximum scavenging value (73.8 ± 0.56%) at 125 μg/mL. However, when the concentration was 250 μg/mL, the DPPH radical scavenging activity of ethanol extract was higher than that of the BHT. The results indicated that the DPPH radical scavenging activity of BHT was higher than that of alcohol extract at low concentration, but the latter was better than BHT at high concentration. Compared with the previous studies, the DPPH radical scavenging activity of the alcohol extract (IC_50_ = 80 µg/ml) from *L. erythrorhizon* (Yang & Zhu, 2007) was lower than that of *OHC*‐LD. Meanwhile, the naphthoquinone compounds have been proven to exhibit excellent antioxidant capacity (Ordoudi et al., 2011). However, in the early report, the contents of naphthoquinones of *L. erythrorhizon* are higher than that of *OHC*‐LD (Hu, Jiang, Leung, & Zhao, 2006). It is referred that the increase of naphthoquinone content in alcohol extract under the optimal condition leads to the enhancement of DPPH radical scavenging ability.

#### 3.2.2 The radical scavenging activity against ABTS

As an excellent substrate for peroxidases, ABTS is frequently used to study the antioxidant properties of natural compounds (Zaidi, Shah, Parmar, & Thawani, 2018). From the Figure 3b, although the ABTS radical scavenging activity of the ethanol extract (IC_50_ = 295.81 µg/ml) was lower than that of BHT (IC_50_ = 32.423 µg/ml), it also could reach to nearly 100 ± 0.79%, when the concentration higher than 1,250 µg/ml. The good ABTS radical scavenging activity may derive from the large number of hydroxyl groups in naphthoquinone molecules (Zaidi et al., 2018), which can provide electrons to decrease ABTS free radical.

#### 3.2.3 Reducing power

The reducing power is another useful method for assessing antioxidant activity. It is indicated by the absorbance quantity for the reaction mixture at 700 nm (Gharibzahedi, Razavi, & Mousavi, 2013). Figure 3c indicated the reducing power values of the ethanol extract increased in a concentration‐dependent manner, but lower than that of BHT. The reducing power value of the ethanol extract reached 1.17 ± 0.03 at 10 mg/ml, while the BHT reached the maximum value (1.2 ± 0.01) at 5 mg/ml.

In general, the optimal extract exhibited similar scavenging activity against ABTS radical as BHT at 1,250 µg/ml, better DPPH scavenging activity than BHT at 250 µg/ml, and similar reducing power as BHT after 10 mg/ml. The existence of numerous hydroxy groups in naphthoquinone molecules could be attributed to antioxidant capacity (Belhaj et al., 2017).

### 3.3 Evaluation of the anticancer activity of the ethanol extract in vitro

The optimal ethanol extract from *OHC*‐LD was evaluated the cytotoxity against MCF‐7 cell lines. Compared with the Adriamycin group (IC_50_ at 0.194 µg/ml), the ethanol extract exhibited a moderate inhibition potency against MCF‐7 cell line, with IC_50_ value of 321.849 µg/ml. It referred that the ethanol extract from the roots of *Onosma hookeri* Clarke. var. longiforum Duthie was not particularly sensitive to MCF‐7 cell lines.

## 4 CONCLUSION

In this study, an efficient and economical ultrasonic‐assisted extraction of ethanol extract from the roots of *OHC*‐LD was established by BBD‐RSM. The optimal conditions were obtained (69% ethanol, material to solution ratio of 27:1 at 60℃ for 59 min). The yield of ethanol extract was 42.08 ± 0.65%, and the content of naphthoquinones reached to 1.07 ± 0.004%. Furthermore, the naphthoquinones‐enriched extract obtained under the optimal condition exhibited better DPPH scavenging activity, similar ABTS radical scavenging effects and reducing power as BHT at high concentrations (250 µg/ml, 1,250 µg/ml, 10 mg/ml, respectively), indicating its good antioxidant activity, comparable to or better than BHT. However, the optimal ethanol extract was not particularly sensitive inhibitory effect against MCF‐7 cells. Under the optimal conditions, the extraction yield was the highest, with a higher content of naphthoquinones, both of which led to the excellent antioxidant capacity of the ethanol extract. Therefore, the naphthoquinones‐enriched ethanol extract from the roots of *OHC*‐LD can be used as an alternative antioxidant agent in different field, including pharmaceutical formulations, cosmetic, and food in the future. Further studies on the composition analysis and other biological activity tests will be investigated in the future.
